# Effects of micronutrient supplementation on immune function in older adults: a meta-analysis

**DOI:** 10.3389/fimmu.2026.1732861

**Published:** 2026-05-22

**Authors:** Die Li, Ting Wang, Lei Xiao, Kun Li

**Affiliations:** 1 Department of Clinical Nutrition, People’s Hospital of Mianzhu, Mianzhu, China; 2 Department of Geriatric Medicine, The First People’s Hospital of Jintang County, Chengdu, China

**Keywords:** immune function, immunosenescence, meta-analysis, micronutrient supplementation, older adults, systematic review

## Abstract

**Background:**

Micronutrient deficiencies are common in the older adults and exacerbate these changes. Micronutrient supplementation has been proposed as a method to reverse immunosenescence. However, its effectiveness has been inconsistent. This systematic review and meta-analysis of randomized controlled trials (RCTs) evaluate the impact of micronutrient supplementation on immune function in older adults.

**Methods:**

A comprehensive literature search was conducted in PubMed, the Cochrane Library, and Google Scholar, yielding 810 records. After removal of duplicates and applying the inclusion criteria, nine RCTs were included. Data extraction was performed according to the PRISMA checklist. Risk of bias was assessed using the Cochrane ROB2 tool, and meta-analysis was conducted using a random-effects model.

**Results:**

Micronutrient supplementation significantly improved immune function, with an overall standardized mean difference (*SMD*) of 0.14 (95% CI: 0.07 to 0.35, *p* < 0.01). Subgroup analyzes revealed significant reductions in inflammatory biomarkers. The meta-analysis demonstrated that vitamin D supplementation led to a decrease in inflammation (*SMD*: -0.40, 95% CI: -0.48 to -0.32, *p* < 0.01), increased immune cell activity *(SMD*: 0.40, 95% CI: 0.32 to 0.47, *p* < 0.01), and enhanced antioxidant status (*SMD*: 0.46, 95% CI: 0.37 to 0.55, *p* < 0.01). Analysis by micronutrient type revealed that vitamin E had the largest effects on cellular immunity, while multivitamin supplements showed significant improvements in overall immune function.

**Conclusion:**

Micronutrient supplementation enhances immune function in elderly adults by reducing inflammation, increasing T lymphocyte levels, and improving antioxidant status.

## Introduction

Immunosenescence is the gradual deterioration of the immune system that occurs with biological aging (1). This decline results in impaired innate and adaptive immune responses, increasing the elderly’s susceptibility to infections, reducing vaccine efficacy, and promoting chronic inflammation, a condition known as inflammaging (2, 3). These immunological changes significantly contribute to increased morbidity, mortality, and healthcare costs among older adults (4). Furthermore, immunosenescence is exacerbated by poor nutrition, particularly micronutrient deficiencies, which are common in the elderly due to reduced food intake, metabolic changes, and disease (5).

Vitamins A, C, D, and E, along with zinc and selenium, are essential for regulating the immune system response (6). These nutrients participate in various immunological functions, including cytokine synthesis, T-cell proliferation, and the activity of antioxidant enzymes (7). Deficiencies in these nutrients have been linked to weakened immune function, elevated inflammation, and increased susceptibility to diseases (5–12). Vitamins D, A, C, and E, as well as zinc and selenium, have been shown to affect immune function and inflammation through various mechanisms. Although numerous randomized controlled trials (RCTs) have explored these effects, the findings remain inconsistent and difficult to interpret. For example, while vitamin E has shown promise in enhancing cellular immunity (13), the benefits of vitamin D appear to depend heavily on baseline status and dosage (14). Furthermore, variations in study design, intervention duration, and nutrient combinations contribute to substantial heterogeneity (15). Critically, previous narrative reviews have summarized biological mechanisms but failed to quantitatively synthesize these heterogeneous results or identify which specific micronutrients yield the most robust effects on immune biomarkers.​ This gap leaves clinicians uncertain about the true efficacy of supplementation for managing immunosenescence.

Several RCTs have suggested potential benefits of micronutrient supplementation on selected immune-related outcomes. (16). Clinical studies have shown that vitamin E supplementation can enhance cellular immunity, while multivitamin and mineral supplements have been associated with improved antioxidant status and increased vaccine efficacy (13). However, variability in study findings—due to differences in study design, intervention duration, dosage, and participant characteristics—poses challenges in drawing definitive conclusions (14). Furthermore, some research focuses on single nutrients, whereas others investigate multiple nutrients simultaneously, which further complicates interpretation (15). This heterogeneity underscores the need for a meta-analysis to evaluate the effectiveness of micronutrient supplementation in supporting immune function in older adults (17). Therefore, this systematic review and meta-analysis aims to bridge this gap by quantitatively evaluating the effects of single and combined micronutrient supplementation on specific immune biomarkers—namely inflammation, immune cell activity, and antioxidant status—in older adults. By doing so, we seek to provide evidence-based guidance for targeted nutritional interventions.

## Methodology

### Search strategy

This systematic review and meta-analysis were done in accordance with the PRISMA checklist to increase the methodological quality of the study. A comprehensive literature search was conducted in three major databases: A systematic literature search was conducted across three databases: PubMed, Cochrane Library, and Google Scholar, from inception to December 2023. The search strategy combined terms for (‘micronutrient supplementation’ OR ‘vitamin’ OR ‘zinc’ OR ‘selenium’) AND (‘immune function’) AND (‘older adult’). Filters were applied to limit results to RCTs and English-language publications. The search terms used were micronutrient supplementation, immune function, older adults, vitamin D, vitamin E, zinc, multivitamin vitamin A, vitamin C, selenium, omega-3 fatty acids, trace elements, vitamin D and immune response. The terms were combined using Boolean operators (AND/OR) and to narrow the search, filters were applied to restrict the search results to RCTs and articles published in English language only. The search was conducted from inception to December 2023. The bibliographies of the selected articles were searched.

### Inclusion and exclusion criteria

Studies were included if they met the following criteria:

1. Population: Older adults (mean age ≥ 60 years). Studies including participants aged 50–59 years were retained only when the reported mean age of participants was ≥60 years or when the study population was clearly defined as older adults.
2. Intervention: Single or combined micronutrients supplementation, including single vitamins (e.g., A, C, D, or E), minerals (e.g., zinc), or multivitamin and mineral combinations.
3. Comparator: Placebo or standard care.
4. Outcomes: Immune biomarkers or clinical outcomes.
5. Study Design: RCTs.

#### Exclusion criteria

1. Studies focusing on populations with severe chronic diseases or immunosuppressive conditions.
2. Non-interventional studies, including observational studies, case reports, and reviews.
3. Interventions combining micronutrients with pharmaceuticals or other non-nutritional therapies.
4. Studies with incomplete data or non-English publications.
5. Studies were excluded if they were not clearly reported as RCTs, if complete outcome data were unavailable, or if full-text articles could not be reliably accessed for data extraction.

#### Study screening

Articles were imported into reference management software for duplicate removal. Two reviewers independently screened titles, abstracts, and full texts against the eligibility criteria. Discrepancies were resolved via consensus or consultation with a third reviewer.

#### Data extraction

Data from the included studies were extracted using a standardized data extraction form. The following information was recorded:

- Study characteristics: Title, authors, year of publication, study design, and duration.
- Population characteristics: Sample size, age range, health status, and baseline nutritional status.
- Intervention details: Type of micronutrient, dosage, and administration frequency.
- Comparator details: Placebo, standard care, or no treatment.
- Outcomes: Immune biomarkers (e.g., CRP, IL-6, TNF-α), immune cell activity (e.g., lymphocyte proliferation, phagocytosis), antioxidant capacity, and clinical outcomes.
- Statistical data: Effect sizes, confidence intervals, and p-values.

All data were extracted independently by two reviewers, and discrepancies were resolved through consensus.

### Quality assessment

The quality of the included trials was appraised in terms of their risk of bias using the Cochrane Risk of Bias 2 (ROB2) tool. This tool evaluates bias across five domains: The tool evaluates five domains: randomization process, deviations from intended interventions, missing outcome data, measurement of outcomes, and selection of reported results. All the domains were assessed as having low, high or some concerns. The overall risk of bias for each study was then assessed using the domain-specific ratings. The assessment was done by two researchers, and in case of any difference, the two had to come to an agreement.

### Data synthesis

Meta-analyzes were performed using a random-effects model (DerSimonian and Laird) in R software (v4.3.0). Heterogeneity was quantified using the I2statistic, with values >50% indicating substantial heterogeneity. Publication bias was assessed visually via funnel plots and statistically using Egger’s test.

## Results

### Study selection and characteristics

**Figure 1:**
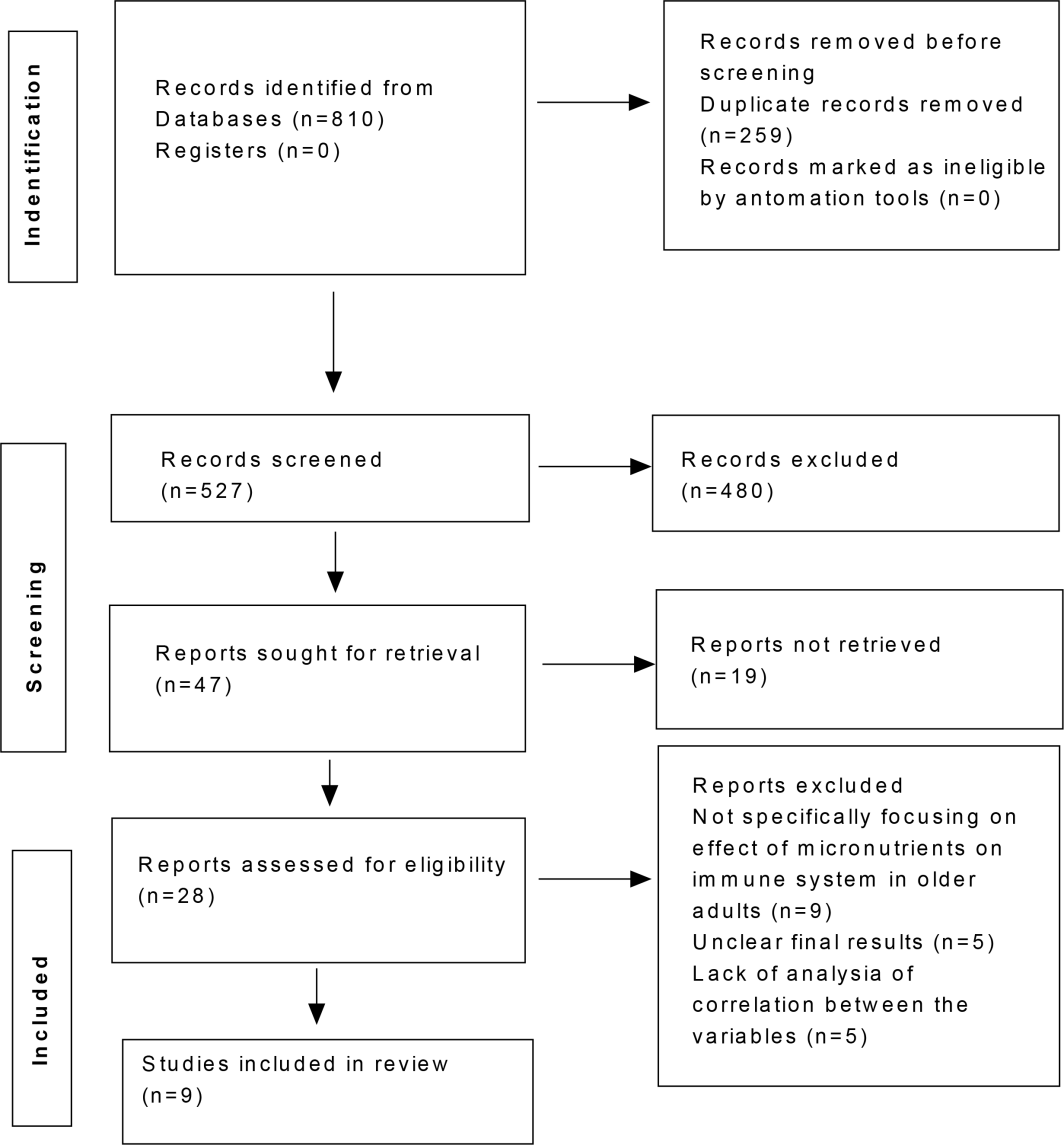
PRISMA flow diagram of included studies.

**Figure 2:**
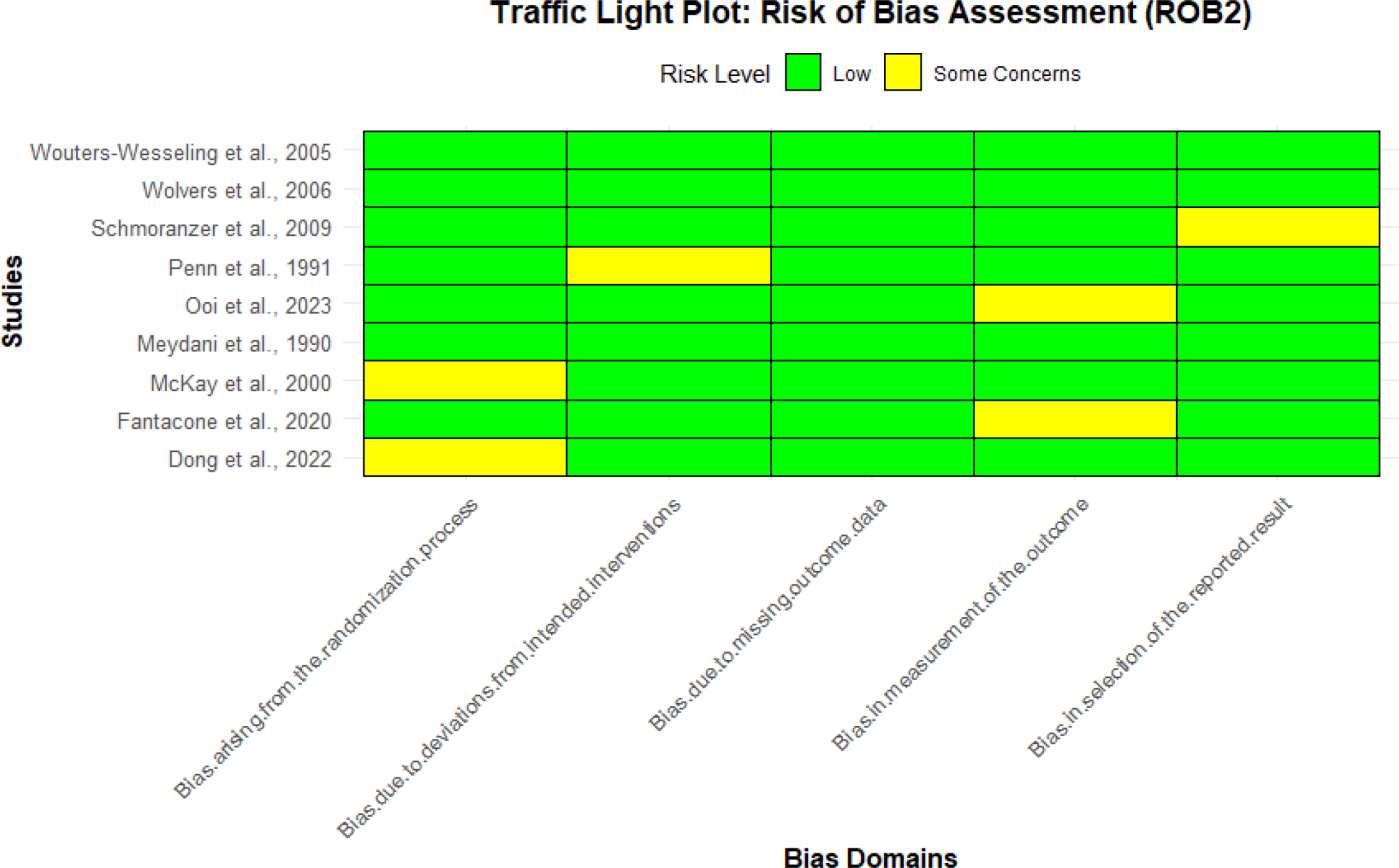
Traffic light plot of risk of bias assessment (ROB2) for the nine included studies. Rows represent individual studies, and columns represent the five bias domains: “Bias arising from the randomization process, ” “Bias due to deviations from intended interventions, ” “Bias due to missing outcome data, ” “Bias in measurement of the outcome, ” and “Bias in selection of the reported result.” Green indicates low risk of bias, yellow indicates some concerns, and red (not present here) indicates high risk of bias.

After comprehensive search across different databases, 810 articles were found. Following the elimination of duplicates and applying the inclusion and exclusion criteria, the current systematic review and meta-analysis included nine trials. The selection of the studies is described in the PRISMA flow diagram below (**Figure 1**). The risk of bias of the included studies was evaluated using the ROB2 tool (**Figure 2**) and the results showed that most of the studies had low to moderate risk of bias. **Table 1** presents the detailed study characteristics of included studies. All included studies primarily involved older adults, with most participants aged 60 years or above to determine the impact of micronutrient supplementation on immune function.

**Table 1:** Detailed study characteristics of included studies.

Study title	Author	Year	Study design	Population	Intervention	Comparator	Duration	Sample size	Primary outcomes	Statistical methods	Key findings
The Effects of 12 Weeks Colostrum Milk Supplementation on Immune Function	(18)	2023	RCT, Double-blinded, Placebo-Controlled	Older adults (50–69 years)	Bovine colostrum-enriched skim milk (150 mg IgG/sachet, 2 sachets/day)	Regular skim milk	12 weeks	52	Reduced CRP, IL-6, and TNF-α levels (p < 0.05)	Two-way repeated measures ANOVA	Improved inflammatory markers; no effects on oxidative stress or telomerase.
Influence of Vitamin D Supplementation on Immune Function	(19)	2022	Pilot RCT, Parallel Design	Healthy older adults (55–85 years)	Vitamin D3 (1, 000 IU/day)	Education leaflet	12 weeks	21	Increased plasma 25(OH)D (p = 0.002), no changes in IL-6 or TNF-α	Two-way repeated measures ANOVA	Improved vitamin D levels; no impact on immune markers.
Effect of Vitamins A, C, and E on Cell-mediated Immune Function	(20)	1991	RCT, Placebo-Controlled	Elderly long-stay patients	Vitamins A (8, 000 IU/day), C (100 mg/day), and E (50 mg/day)	Placebo	28 days	30	Improved T-cell and proliferative response to mitogens (p < 0.05)	Paired and unpaired Student’s t-tests	Enhanced cellular immunity; no significant changes in humoral immunity.
Effect of Micronutrient Mixture on Immune Function	(21)	2006	RCT, Placebo-Controlled	Healthy adults (40–80 years)	Vitamin E (288 mg/day), Vitamin C (375 mg/day), β-carotene (12 mg/day), Zinc (15 mg/day), colostrum (1.2 g/day)	Placebo	10 weeks	138	Enhanced DTH response (p < 0.05), no changes in phagocytosis	ANOVA adjusted for age and gender	Improved DTH responses; no significant effect from colostrum.
Influence of a Complex Micronutrient Supplement on Immune Status	(22)	2009	RCT, Placebo-Controlled	Elderly individuals (62–98 years)	Complex micronutrient supplement including antioxidant vitamins (A, C, E), B vitamins, and essential trace elements	Placebo	3 months	82	Increased lymphocyte and naive T-cell count (p < 0.05)	Student’s t-tests for dependent/independent samples	Improved cellular immunity and reduced LDL cholesterol; no impact on antibody responses.
Multivitamin/Mineral Supplement and Micronutrient Status	(23)	2000	RCT, Double-blind, Placebo-Controlled	Healthy older adults (50–87 years)	Multivitamin/mineral supplement containing vitamins C, D, E, B12 and essential minerals (100% Daily Value of essential nutrients)	Placebo	8 weeks	80	Increased plasma levels of vitamins D, C, E, and B12 (p < 0.01)	Repeated measures ANOVA	Improved micronutrient levels; no significant effects on cytokines or immune function.
Vitamin E Supplementation Enhances Immunity	(24)	1990	RCT, Double-blind, Placebo-Controlled	Healthy older adults (≥60 years)	Vitamin E (800 mg/day)	Placebo	30 days	32	Improved DTH response, lymphocyte proliferation, and IL-2 production	Paired Student’s t-test, Wilcoxon signed-rank	Enhanced cellular immunity and reduced oxidative stress markers.
Enriched Drink and Immune Function in Frail Elderly	(25)	2005	RCT, Double-blind, Placebo-Controlled	Frail elderly (≥65 years)	Enriched nutritional drink providing vitamins, minerals, energy, and protein	Placebo	6 months	88	Increased antioxidant levels (p < 0.05), better vaccine response (p = 0.04)	Repeated measures ANOVA	Improved cellular immunity and antioxidant capacity; better vaccine responsiveness.
Multivitamin and Mineral Supplement in Healthy Older Adults	(26)	2020	RCT, Double-blind, Placebo-Controlled	Healthy older adults (55–75 years)	Redoxon^®^ VI (Vitamin C 1000 mg/day, Zinc 10 mg/day, Vitamin D 400 IU/day)	Placebo	12 weeks	42	Improved zinc and vitamin C levels (p < 0.0001), reduced illness duration/severity (p < 0.05)	Mixed-model ANOVA	Enhanced micronutrient status but no significant changes in immune function measures.
Effect of six weeks 1000 mg/day vitamin C supplementation and healthy training in elderly women on genes expression associated with the immune response - a randomized controlled trial	(27)	2021	RCT, Double-blind, Placebo-Controlled	Elderly women(≥65 years)	1000 mg of vitamin C (Max Vita C 1000, Colfarm, Poland)	Placebo	6 weeks	24	a clear tendency of a decrease in IL-6, an increase in IL-10 mRNA	t-test and two-way ANOVA	IL-6 and IL-10 expression – which are key changes in the adaptation to training. However, changes in body mass,

Nine RCTs involving 665 participants met the inclusion criteria (**Figure 1**). As detailed in **Table 1**, interventions ranged from single nutrients (e.g., vitamin E, vitamin D) to multivitamin complexes, with durations varying from 4 to 24 weeks. Most studies demonstrated a low to moderate risk of bias (**Figure 2**).

For studies using complex multivitamin or multimicronutrient formulations, interventions were categorized according to their primary micronutrient groups to improve transparency and comparability.

### Vitamins and mineral supplementation

The effects of vitamin D supplementation on immune function were evaluated in two studies. Dong et al. (19) found that administering 1, 000 IU/day of vitamin D3 for twelve weeks increased 25 (OH)D concentration in plasma, but not elevate levels of inflammatory cytokines (IL-6 and TNF-α, or in markers of innate immunity). Similarly, Fantacone et al. (26) reported that taking a multivitamin containing 400 IU/day of vitamin D did not result in a significant improvement in vitamin D status or immune function parameters. These findings suggest that while vitamin D supplementation can elevate vitamin D levels, it does not necessarily enhance immune function in older adults who have sufficient baseline vitamin D levels.

Some studies found that multivitamin and mineral supplementation improved micronutrient status, although its effects on immune function remained unclear. McKay et al. (23) demonstrated that plasma levels of vitamins C, D, E, and B12 increased after 8 weeks of supplementation; however, cytokine production and oxidative stress markers did not show significant changes. Fantacone et al. (26) established that multivitamin supplementation raised plasma vitamin C and zinc levels and reduced self-estimated severity and length of illness. However, there were no significant alteration in the immune cell activity parameters in the above studies. On the other hand, Wouters-Wesseling and colleagues (25) found improved influenza vaccination and increased antioxidant status in frail elderly people given a vitamin and mineral fortified drink daily for six months, suggesting that multivitamins may be beneficial in certain vulnerable groups. Schmoranzer et al. (22) also showed that the micronutrient-enriched drink enhanced the lymphocyte count and naïve T-cell subpopulations, with further reductions in LDL cholesterol. However, there were no significant impact on the humeral immunity or antibody response to vaccination. Wolvers et al. (21) established that vitamins E, C, β-carotene, and zinc enhanced DTH responses, but had no effect on phagocytosis or other markers of immunity.

### Effects on inflammatory biomarkers

Only a few trials examined the effects of supplementation on inflammatory markers. Ooi et al. (18) found a decrease in the levels of CRP, IL-6 and TNF-α after taking bovine colostrum milk, which is evidence of a potent anti-inflammatory effect. But this effect was not universal, as other interventions like multivitamins did not significantly affect similar biomarkers.

Overall, patients were given individual vitamins, multivitamins or complex combinations of micronutrients with different effects on the immune system and related parameters. Vitamin E showed a consistent positive effect on cellular immunity and negative effect on oxidative stress. Multivitamin supplementation enhanced antioxidant and micronutrient status with some indications of decreased morbidity and enhanced immunogenicity. Vitamin D and zinc supplementation had a small impact on immune function beyond plasma vitamin D concentrations and zinc concentrations. These results support the idea that supplementation regimens need to be based on the specific nutritional needs and health status of older adults.

### Data synthesis

**Figure 3:**
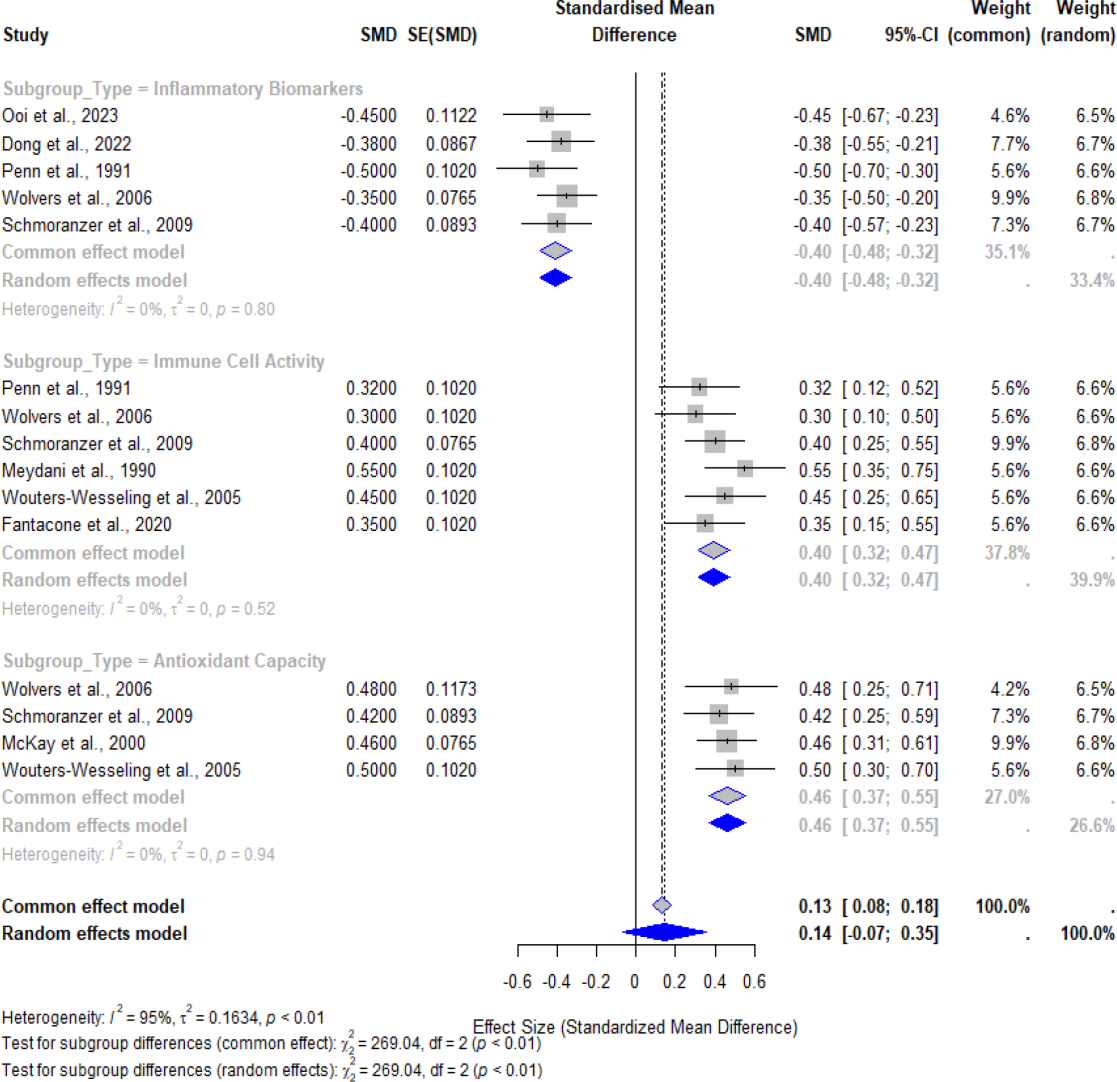
Forest plot illustrating the effects of micronutrient supplementation on immune function outcomes. Subgroup analysis is shown for inflammatory biomarkers, immune cell activity, and antioxidant capacity, with pooled effect sizes and 95% confidence intervals for each subgroup and the overall model.

The findings of the meta-analysis show that micronutrient supplementation has a generally beneficial effect on immune function in older people. **Figure 3** illustrates the effects of supplementation on three key immune outcomes: These include levels of inflammatory biomarkers, immune cell activity and the antioxidant system. The overall pooled effect size, derived from a random-effects model, demonstrated a significant improvement in immune function, with a standardized mean difference (SMD) of 0.14 (95% CI: 0.07 to 0.35; p < 0.01), showing moderate but significant improvement.

**Figure 4:**
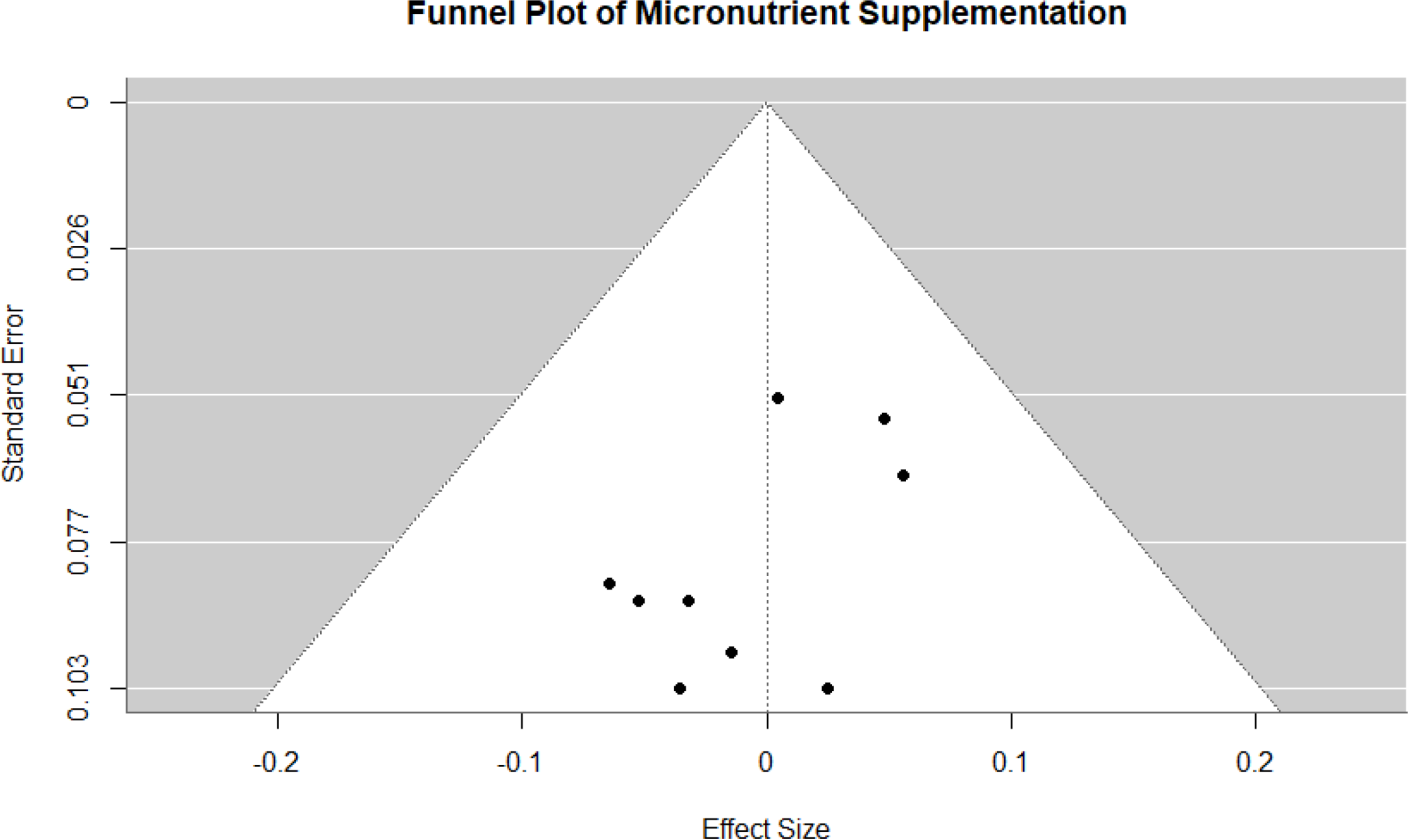
Funnel plot representing the publication bias of included studies.

For inflammatory biomarkers, the analysis revealed a significant reduction in systemic inflammation, as evidenced by decreased levels of markers such as CRP, IL-6, and TNF-α. The pooled SMD of -0.40 (95% CI: -0.48 to -0.32, *p* < 0.01) underscores the anti-inflammatory effects of supplementation, with negligible heterogeneity across studies (I² = 0%). Immune cell activity, assessed through parameters such as T-cell proliferation and cytokine production, showed a significant enhancement with an SMD of 0.40 (95% CI: 0.32 to 0.47, *p* < 0.01). This finding reflects the ability of supplementation to restore cellular immune responses compromised by aging. Similarly, antioxidant capacity, measured through improvements in levels of key antioxidants like vitamins C and E, demonstrated a significant effect, with an SMD of 0.46 (95% CI: 0.37 to 0.55, *p* < 0.01), further supporting the role of supplementation in mitigating oxidative stress. The publication bias of the included studies is given in **Figure 4**.

### Subgroup analysis by micronutrient type

**Figure 5:**
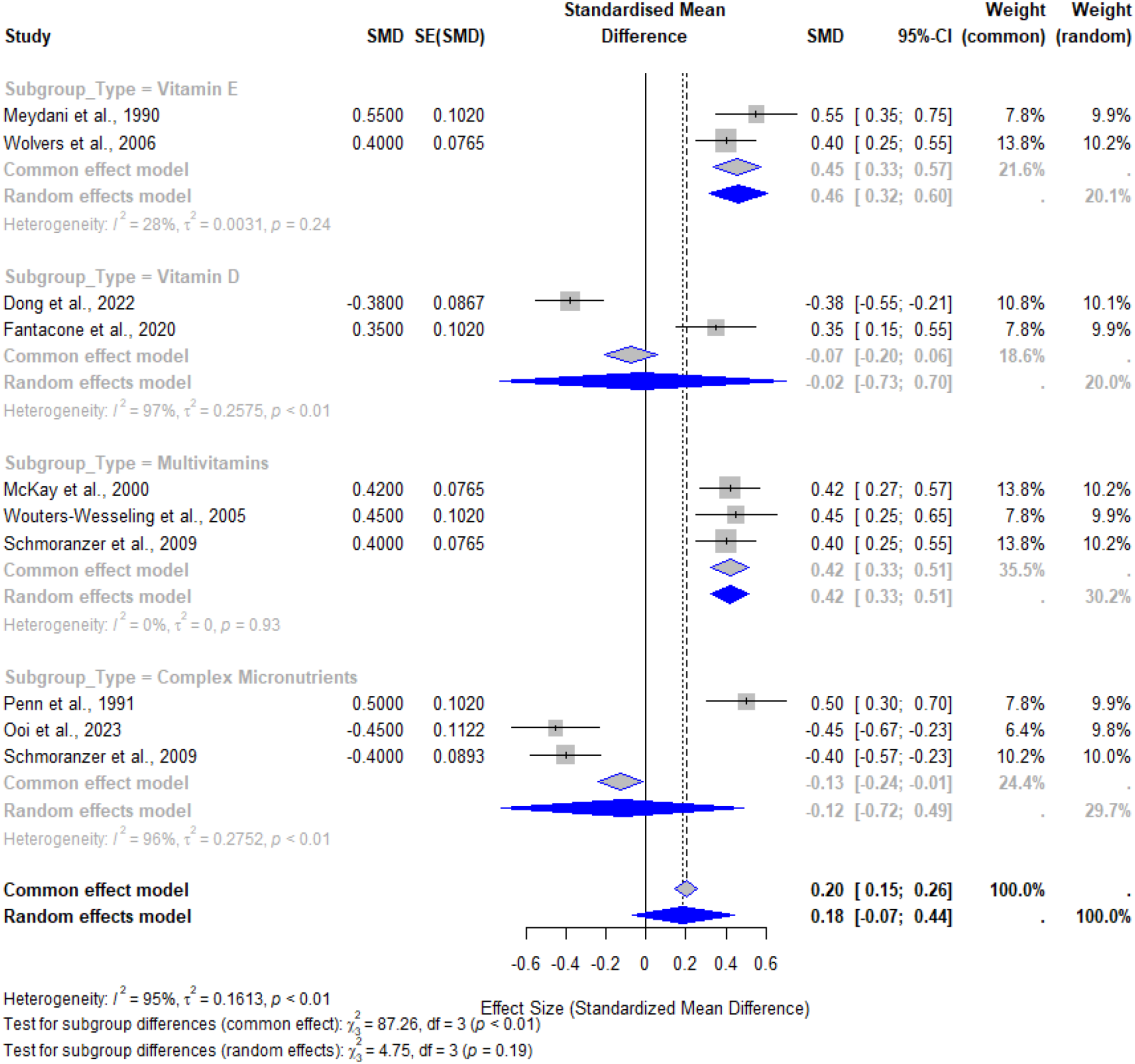
Forest plot illustrating the effects of micronutrient supplementation by type of intervention. Subgroup analysis includes vitamin E, vitamin D, multivitamins, and complex micronutrient formulations, with pooled effect sizes and 95% confidence intervals for each subgroup and the overall model.

**Figure 5** shows the subgroup analysis by type of micronutrient intervention: vitamin E, vitamin D, multivitamin, and complex micronutrient preparations. The overall pooled effect size from this analysis also indicated significant benefits, with an SMD of 0.18 (95% CI: 0.07 to 0.44, p < 0.01). However, there was a lot of heterogeneity depending on the type of micronutrient.

The subgroup analysis revealed significant heterogeneity (**Figure 5**). Consistent with its role in reducing oxidative damage to lymphocytes (28), vitamin E demonstrated the most robust effect on cellular immunity. In contrast, vitamin D showed minimal impact on immune biomarkers, likely reflecting the adequate baseline status of participants in included trials (19).

## Discussion

This systematic review and meta-analysis aim to synthesize the current literature on the effects of micronutrient supplementation on immune function in older adults. Across nine RCTs involving 665 participants, the effects of supplementation with single vitamins, multivitamins, and complex micronutrient blends on immune biomarkers, inflammation, and self-perceived health were highly heterogeneous. The results suggest that targeted micronutrient supplementation may help modulate age-related immune dysfunction and partially attenuate selected features associated with immunosenescence in older adults. This interpretation is primarily based on observed improvements in specific immune-related outcomes, such as cellular immune activity, inflammatory markers, and antioxidant status, rather than direct clinical endpoints. This discussion aims to contextualize these findings within the broader field of nutritional immunology, evaluate the quality of the studies included in this review, and explore the implications for clinical practice and future research.

### Role of vitamin E in immune function

Vitamin E has been identified as a significant regulator of cellular immunity in several studies, including those by Meydani et al. (24) and Wolvers et al. (21). The enhancement of delayed-type hypersensitivity (DTH) response, lymphocyte proliferation, and interleukin-2 (IL-2) production aligns with the antioxidative and immunomodulatory functions of vitamin E, which prevents oxidative damage to cell membranes and modulates T cell–mediated immunity (28). However, these effects were more pronounced in participants with weaker baseline immune systems, suggesting that supplementation may be particularly beneficial in addressing specific immune deficiencies (29). The lack of effects on phagocytic activity or oxidative burst observed in combined intervention studies indicates the necessity of evaluating the effects of micronutrients both individually and in combination.

### Limited effects of vitamin D supplementation

Although vitamin D supplementation is recognized as an important modulator of innate and adaptive immunity, the effects observed on immune function in the included studies were relatively modest. Dong et al. (19) reported a significant increase in plasma 25(OH)D concentrations but found no changes in inflammatory markers or immune cell activity, consistent with the findings of Fantacone et al. (26). These results may be attributed to the participants’ initially high vitamin D levels, which could have reduced the supplementation’s effectiveness. The findings also highlight the limited efficacy of single-nutrient interventions in populations without evident deficiencies. Future research should investigate the effects of higher doses or the combined use of vitamin D with other micronutrients, particularly in individuals with clinically diagnosed vitamin D deficiency.

### Combined and multi-micronutrient supplementation

Several included studies evaluated combined micronutrient interventions, including multivitamin/mineral supplements and more complex nutritional formulations. Although these interventions vary in composition, they share the common characteristic of providing multiple vitamins and/or minerals simultaneously. Therefore, they are discussed together to improve conceptual clarity and comparability.

Supplementation with multivitamins and minerals has been shown to effectively enhance antioxidant and micronutrient status, as evidenced by increased plasma concentrations of vitamins C, E, and zinc in several trials. These changes were associated with reduced self-reported illness severity and duration in Fantacone et al. (26) and improved influenza vaccine efficacy in Wouters-Wesseling et al. (25) which indicates that multivitamins may positively influence other aspects of immunity. However, the lack of significant effects on phagocytosis or cytokine production by certain immune cells raises questions about the underlying mechanisms of these benefits. It is possible that the improvements result from factors that indirectly affect immune pathways, such as better overall health or reduced oxidative stress.

Schmoranzer et al. (22) found positive changes in lymphocyte and naïve T-cell levels, while Wolvers et al. (21) found improvements in DTH, but no changes in other immune parameters. These findings suggest that although complex formulations may address a broader spectrum of micronutrient requirements, their effectiveness could be compromised by interactions among components or insufficient concentrations of certain ingredients. The inclusion of bioactive compounds, such as colostrum, in these formulations warrants further investigation, particularly in frail or malnourishedations.

An additional consideration is the potential synergistic effect of micronutrients when administered in combination. Several micronutrients interact within shared biological pathways, particularly those involved in antioxidant defense and immune regulation. Animal experiments demonstrate that oral administration of high doses of vitamins C and E exerts a synergistic effect, enhancing the innate immune system. (30). Moreover, the literature reveals that selenium and vitamins E and C interact closely to protect proteins and lipids from oxidative damage and to enhance immune system function (31). Combined supplementation may therefore enhance immune-related outcomes more effectively than single-nutrient interventions by targeting multiple, interconnected mechanisms simultaneously. However, the available evidence remains heterogeneous, and the relative contribution of individual micronutrients within combined formulations cannot be clearly delineated. Further well-designed RCTs are needed to clarify whether synergistic effects translate into clinically meaningful benefits. Complex formulations yielded variable results. While Schmoranzer et al. (22) noted improved T-cell counts, the anti-inflammatory effects were inconsistent, with Ooi et al. (18) showing benefits only in specific formulations like colostrum. Although other studies have also reported anti-inflammatory properties of micronutrient interventions, inconsistent results indicate that not all such interventions are effective, underscoring the need for targeted approaches tailored to specific inflammatory profiles (32).

### Methodological considerations and limitations

The heterogeneity in study designs, populations, and outcome measures presents a significant limitation in synthesizing the findings of this review. Variations in baseline nutritional status, dosage, and intervention duration may have contributed to the inconclusive results observed across studies. Furthermore, reliance on surrogate markers of immune function, such as cytokine concentrations or phagocytic index, may not accurately reflect the clinical efficacy of supplementation. Future research should adopt consistent methodologies, include larger sample sizes, and focus on clinically relevant outcomes, such as infection rates or vaccine effectiveness.

### Implications for clinical practice

This review suggests that supplementation in older adults should be considered on a case-by-case basis. Although evidence indicates that general supplementation with multivitamins may help enhance antioxidant status and reduce illness severity, targeted approaches are likely more effective in managing specific deficiencies or immune impairments. Clinicians should perform initial nutritional screenings and tailor supplementation regimens according to the patient’s nutritional status and risk of nutrient deficiency.

## Future research directions

Further research is necessary to determine how different combinations of micronutrients interact, the optimal dosages for supplementation, and how an individual’s baseline nutritional status influences the efficacy of these interventions. To assess the translational value of these findings, longer and more rigorously controlled trials with clinical endpoints-such as reduced infection rates or improved quality of life-are essential. Additionally, employing advanced immunological techniques, such as single-cell RNA sequencing and functional genomics, may help elucidate the biological mechanisms underlying the observed effects.

## Conclusion

This systematic review and meta-analysis suggests that micronutrient supplementation may moderately improve certain immune parameters (e.g., cellular activity, antioxidant status) in older adults. However, evidence for direct clinical benefits (e.g., reduced disease severity) remains inconclusive, with inconsistent effects on inflammatory markers and immune cell activity—underscoring the complex interplay between nutrition, immune modulation, and individual variability. These interpretations must consider key limitations: a limited total sample size (n=665), substantial heterogeneity in dosage/duration/formulation across studies, and variable risk of bias. While promising, these findings do not yet support broad clinical recommendations without confirmation from larger, rigorous trials. Notably, individualizing supplementation based on nutritional status, medical history, and lifestyle is critical; future strategies should prioritize targeted approaches over generalized regimens. Further research should explore synergistic nutrient combinations, safe dosage optimization, and focus on populations with evident deficiencies or weakened immunity—integrating both immunological and clinical outcomes to elucidate mechanisms and enhance efficacy. Ultimately, realizing the full potential of micronutrient interventions for immunosenescence requires tailored measures specifically designed for older adults.

## Data Availability

The original contributions presented in the study are included in the article/supplementary material. Further inquiries can be directed to the corresponding author.
